# Antibacterial activity of self-adhesive resin cements against *Streptococcus mutans* at different time intervals

**Published:** 2019-08

**Authors:** Amir-Ahmad Ajami, Sahand Rikhtegaran, Mahmoud Bahari, Sayeh Hamadanchi

**Affiliations:** 1 Department of Operative Dentistry, Faculty of Dentistry, Tabriz University of Medical Sciences, Tabriz, Iran; 2 Dental and Periodontal Research Center, Faculty of Dentistry, Tabriz University of Medical Sciences, Tabriz, Iran

**Keywords:** Anti-bacterial agents, Resin cements, Self-etch primer, Dental caries, Bacteria

## Abstract

**Background and Objectives:**

Self-adhesive resin cements release fluoride and have cytotoxic and preventive monomers against the bacteria in their composition. They have acidic property before their complete setting too. The antibacterial activity of three different self-adhesive resin cements against *Streptococcus mutans* at different time intervals was investigated in this study.

**Materials and Methods:**

The modified direct contact test was used to evaluate the antibacterial effect of Max-Cem, G-Cem and Bis-Cem on *S. mutans* after aging the samples in phosphate-buffered saline solution for one hour, 24 hours and 1 week. Data were analyzed using one-way ANOVA, repeated measurement ANOVA and Tukey HSD tests (P<0.05).

**Results:**

The differences in the mean bacterial counts between all the study groups and between the study groups and the corresponding control groups were significant at 1-hour and 24-hour intervals (P<0.001). At 1-week, only the differences between Bis-Cem and G-Cem, between Max-Cem and Bis-Cem, and between Bis-Cem and the corresponding control group were significant (P<0.001). There were significant differences between G-Cem and Max-Cem at all the time intervals (P<0.001). In addition, with the use of Bis-Cem there were significant differences between 1-hour and 1-week (P=0.01) and 24-hour and 1-week (P<0.001).

**Conclusion:**

All the cements exhibited antibacterial activity after 1 hour and 24 hours. However, after 1 week, only Bis-Cem retained its antibacterial activity.

## INTRODUCTION

Nowadays, because of some restrictions of the direct restorations such as high wear, polymerization shrinkage and long chair-side time, indirect restorations have been considered. With the use of these kinds of restorations, there is also a chance of fluids leak and microleakage after cementation, which is considered a common clinical phenomenon. Subsequently, oral liquids, ions, molecules and oral bacteria percolate between the tooth and restorative interface. Then an appropriate space for the growth of these cariogenic bacteria, especially *Streptococcus mutans (S. mutans)*, will be created, resulting in recurrent caries. In order to prevent this detrimental effect, it is of vital importance to choose a proper cement (1).

Cements that are used in indirect restorations are divided, based on the dominant setting reaction type, into types that include acid-base reaction (Glass Ionomer, Resin modified Glass Ionomer, Zinc Oxide Eugenol, Zinc Poly Carboxylate and Zinc Phosphate) and resin types with their setting is done with polymerization (2). Previous studies revealed that conventional acid-base cements have limited or some amount of antibacterial activity against *S. mutans*. While incorporating chlorhexidine/Certimide mixtures to their formulation may provide greater antibacterial effect (3). According to Feoz et al. (4) Zinc Phosphate and Zinc Oxide Eugenol exhibited highest amount of antibacterial activity. While, Glass Ionomer cement was the weakest of all. Conversely, *in vitro* studies have shown that both conventional and resin-modified glass-ionomers can decrease artificial caries; they can also remineralize carious lesions in vivo (5) and enhance fluoride uptake by underlying dentin (6). This property might be as attributed to the presence of fluoride and zinc in their structure and their initial acidic pH. Both fluoride and zinc exert several effects on dental plaque bacteria, with both inhibiting a variety of enzymes in intact cells; as a result, they are both widely used as antimicrobials in oral hygiene products, predominantly as anticariogenic agents (7).

Currently, adhesive resin cements are used for cementation of indirect restorations. Adhesive resin cements have etch-and-rinse, self-etch and self-adhesive types. In self-adhesive generation, which are the most recent type, all of the etching, bonding and luting steps are carried out in one step and all together (8). This generation has some advantages such as decreasing dissolution in oral liquids, decreasing working time, less working sensitivity and presence of fluoride in its structure (9).

In self-adhesive resin cements, the pH of the self-etching primer is sufficiently low to demineralize the smear layer and the underlying dentin surface so that etching and priming of dentin can be accomplished simultaneously (8). Therefore, the separate acid-etching step is generally omitted. However, due to the non-rinsing procedure, residual bacteria may remain at the interface between the tooth and the restorative material. The dentin primer is the component that comes into contact and reacts with the dentin substrate at the first stage of restoration. Furthermore, if tooth conditioners, such as primers present in the composition of these luting agents, possess antibacterial activity, these bacteria could be eliminated, thereby preventing secondary caries. Thus, the antibacterial activity of these self-etching primers that are directly applied to the dentin plays an important role in the longevity of the restoration (9).

Considering the release of fluoride and the primary low pH and acidic property of the self-adhesive resin cements and the presence of cytotoxic and preventive monomers against the bacteria in their composition, the aim was to assess the antibacterial activity of three self-adhesive resin cements against *S. mutans* at different time intervals.

## MATERIALS AND METHODS

For the purpose of the present study, three different self-adhesive resin cements, including Bis-Cem (Bisco, Schaumburg, IL, USA), G-Cem (GC Corporation, Itabashi-ku, Tokyo, Japan) and Max-Cem Elite (Kerr, Orange, CA, USA) were used as experimental groups. Compositions and manufacturer information for these cements are provided in Table 1.

**Table 1.** Composition and manufacturers of Self-adhesive resin cements

**Resin cement**	**Manufacture**	**Composition**
Max-Cem	Kerr, Orange, CA, USA	TEGDMA, GPDM, inert mineral fillers, ytterbium fluoride, proprietary Redox initiators, activators, Stabilizers
Bis-Cem	Bisco, Schaumburg, IL, USA	BisGMA, dimethacrylates, phosphate acidic monomer, glass filler
G-Cem	GC Corp., Tokyo, Japan	UDMA, fluoroaluminosilicate glass, initiator, stabilizer, 4MET, phosphoric acid ester monomers, water, dimethacrylate, silica powder

TEGDMA; Triethylene glycol dimethacrylate, GPDM; Glycerophosphoric acid dimethacrylate, BisGMA; Bisphenol glycidyl methacrylate, UDMA; Urethane dimethacrylate, 4MET; 4-Methacryloxyethyl trimellitic acid

### Samples grouping.

Since it is necessary for the bacteria floating in the liquid culture media to be exposed to the resin cement, the modified direct contact test was used for the purpose of this study. Based on the results of a pilot study, 58 microplates were used for each experimental group. In addition, 58 microplates with the bacterial solution but without cements (positive control) and 5 microplates with the cements under study but without any bacteria (negative control) were used as the negative control. Another five microplates without cements and bacteria, with only the culture media, was prepared in order to control the sterility of microplates.

### Modified direct contact test (10, 11).

For every experimental group, 1 mm of the height of microplates was filled with the selected cement and polymerization was carried out according to the manufacturer’s instructions. Then the microplates underwent an aging process by storage in phosphate-buffered saline solution at 37°C with 95% atmospheric moisture for 1 hour, 24 hours and 1 week. During the aging process for 1 week, the physiologic serum was refreshed every 24 hours. At the end of the each aging period, the physiologic serum contents of the microplates were retrieved and 10 μL of *S. mutans* bacterial suspension (approximately 10^6^ bacteria) were added to each microplate. The microplates were kept at 37°C for 60 minutes in a moist environment.

During this period, the bacteria came into direct contact with the free surface of the cements. Then 240 μL of Brain Heart Infusion (BHI) culture medium were added to each microplate and mixed for 2 minutes. In the final stage, serial dilutions were prepared from the content of each micro tube in the BHI culture medium and 20 μL of each dilution was cultured on BHI culture plate using the spreading technique. The bacterial counts were described as CFU/mL.

### Statistical analysis.

One-way ANOVA was used to analyze data obtained for each time interval separately. Post-hoc Tukey HSD tests were used for two-by-two comparisons of the groups in cases of significant differences. Furthermore, repeated measurement ANOVA was used to analyze bacterial counts at three time intervals for each group, separately. In this study, P<0.05 was considered statistically significant.

## RESULTS

The means, standard deviations and standard errors of bacterial counts (CFU/mL) in the study groups are presented in Table 2. The results of Kolmogorov-Smirnov test showed normal distribution of data in the study groups (P>0.05). One-way ANOVA showed significant differences in mean bacterial counts between the study groups (P<0.001).

**Table 2.** Mean colony count (cfu/mL) and standard deviations (SD) of *Streptococcus mutans* growth after exposure to three different cements after different time intervals.

**Exposure time**		**G-Cem**		**Max-Cem**		**Bis-Cem**	
		
		**Test**	**Control**	**Test**	**Control**	**Test**	**Control**
1 Hour	Mean	2068.9^Aa^	7424^Ba^	3098.2^Ca^	7453.6^Da^	5459.9^Ea^	7543.8^Fa^
	SD	221.79	524.31	312.59	396.39	717.23	501.57
24 Hours	Mean	6706^Ab^	7550^Ba^	3712.4^Cb^	7181.1^Da^	5495.8^Ea^	7191.6^Fa^
	SD	387.95	379.39	962.92	495.51	458.19	1069.8
7 Days	Mean	7482.6^Ac^	7416.4^Aa^	7399.3^Ac^	7336.7^Aa^	6945.5^Bb^	7256.2^Ca^
	SD	355.95	465.61	391.09	508.12	170.86	476.9

In each row, different upper letters show statistically significant differences between resin cements (p<0.05).

In each column, different lower letters show statistically significant differences between time intervals (p<0.05).

Two-by-two comparisons of the groups with post hoc Tukey tests showed significant differences in mean bacterial counts between all the study groups and between the study groups and the corresponding control groups at 1-hour and 24-hour intervals (P<0.001). At 1-week interval, the differences between Bis-Cem and G-Cem and between Max-Cem and Bis-Cem were significant (P<0.001). In addition, the difference between the Bis-Cem group and the corresponding control group was significant (P<0.001). In other cases, no significant differences were detected between the study groups and between the study and control groups at this time interval (P>0.05).

Repeated measurement ANOVA was used to compare the mean bacterial counts with the use of each cement at different time intervals. The results showed significant differences between G-Cem and Max-Cem cements at all the intervals (P<0.001), with an increase in bacterial counts over time. In this context, there were significant differences in Bis-Cem cement between 1-hour and one-week (P=0.01) and 24-hour and 1-week (P<0.001) intervals; however, the difference between 1-hour and 24-hour intervals was not significant (P>0.05).

## DISCUSSION

Secondary caries is the most common etiologic agent involved in the failure of dental restorations, which is a localized lesion affecting restoration margins, believed to be associated with residual bacteria and microleakage; its etiology and histology is similar to those of primary caries. It is difficult to diagnose secondary caries and it cannot be permanently managed by operative strategies. One technique to decrease the frequency and severity of this issue is to use fluoride-containing restorative materials and luting agents (8).

The agar diffusion test (ADT) was the most frequently used method for the evaluation of antimicrobial activity of various cements. However, its disadvantages semi-quantitative results that rely on solubility and diffusion characteristics of the test material - and the medium used are well recognized (12). Weiss et al. introduced a direct contact test (DCT) that circumvents many of the problems of ADT (13). It is a quantitative assay allowing the use and testing of water-insoluble materials. It uses direct and close contact between the test microorganisms and materials, being almost independent of the diffusion properties of both the tested material and the media. Apart from its reproducibility and quantitative nature, DCT is relatively insensitive to the size of the inoculate brought into contact with the test material, facilitating simultaneous standardized measurements of a large number of specimens and their controls on the same micro-titer plate to monitor the microbial growth, both in the presence and absence of the test material (13). As a result, in this study a modified DCT was used for the assessment of antibacterial properties of three commercially available self-adhesive resin cements (G-Cem, Max-Cem and Bis-Cem) at different time intervals (1 hour, 24 hours, 1 week).

All the cements evaluated in the present study exhibited significant antibacterial activities for after 1 hour and 24 hours compared with the positive control group. Similarly, Magalhaes et al. (14) showed that RelyX ARC a conventional resin cement and RelyX U200 a self-adhesive one exhibit significant antibacterial activity against *S. mutans* for 24 hours. Previous studies have shown that the formation of *S. mutans* colonies significantly decreased at pH values <5.1, being completely inhibited at pH values ≤4.8 (15, 16). The most probable reason for this finding might be the low primary pH of these cements. These cements have an acidic pH value due to the acidic monomers in their structure that are responsible for self-etching capacity (17). According to manufacturers, the self-etching capacity is provided by the presence of different monomers in the luting agent formulation: GPDM in Maxcem, the hydrophilic monomer 4-META and phosphoric acid ester monomers in G-Cem, and phosphoric acid ester monomers in Bis-Cem.

Furthermore, at 1-hour interval, G-Cem exhibited a higher antibacterial activity compared to the other two groups. In addition, Max-Cem exhibited a higher antibacterial activity compared to Bis-Cem. However, after 24 hours, Max-Cem exhibited a higher antibacterial activity than the other two groups and G-Cem showed a higher antibacterial activity than Bis-Cem. According to Han et al., the primary pH after mixing of G-Cem is 1.8, which is lower than those of Max-Cem (pH=2.4) and Bis-Cem (pH=3.6) (6, 17, 18). Furthermore, the pH values 48 hours after polymerization were 2.4 for Max-Cem, 3.6 for G-Cem, and 4.0 for Bis-Cem, corresponding to the antibacterial activities of these cements at 1- and 24- hour intervals, respectively (6, 19).

In contrast, at 1-week interval the antibacterial activity of Bis-Cem was higher than those of G-Cem and Max-Cem are, which exhibited similar antibacterial properties at this time interval. Based on a literature review, the antibacterial activity of restorative materials might also be attributed to the fluoride content. A large number of studies have shown that fluoride ion is safe and effective in preventing and controlling caries at certain doses. Fluoride can inhibit the growth of oral streptococci *in vitro* at a concentration range of 0.16–0.31 mmol/L, also (24, 25).

The composition, solubility and permeability of the resin matrix, and the source, size and concentration of the fluoride ions are important characteristics of the fluoride-releasing materials (26–29). Aguiar et al. (9), reported that Max-Cem released less fluoride in water compared to Bis-Cem. Max-Cem resin cement contains fluoroaluminosilicate particles that bear resemblance to the glass powder in G-Cem. The similarity in the fluoride source of both materials might explain the similarity in antibacterial activity of G-Cem and MaxCem. However, Bis-Cem self-adhesive resin cement contains glass fillers composed of fluoride glass. The glass fillers are supplied in both base and catalyst pastes of Bis-Cem cement, releasing fluoride in water. Furthermore, the glass powder serves as a reservoir for fluoride (30).

As shown in previous studies, all the resin cements released high levels of ion on the initial days for all the resin cements, demonstrating that the release of fluoride was not uniform over time (9, 21, 26, 31). According to Aguiar et al. (9), after 15 days, fluoride release from Bis-Cem was 10 folds greater than its release from Max-Cem, possibly a further evidence of higher anti-bacterial activity of Bis-Cem compared to Max-Cem and G-Cem, which have similar fluoride particles.

A number of different mechanisms are involved in the anti-cariogenic effects of fluoride on teeth. It can act directly or it forms of metal complexes that inhibit many enzymes (32). However, it appears its main action that leads to the inhibition of acid production by intact bacterial cells at low pH is related to its capacity to enhance proton permeability of cell membranes by acting in the form of protonated fluoride (HF) as a trans-membrane proton carrier. Fluoride prevents proton extrusion by F-ATPases by returning excreted proton back into the cell through movements of HF; the cell is approximately 10 times more permeable to HF compared to fluoride. HF in the relatively alkaline cytoplasm dissociates to yield the enzyme poison F and H1, which acidifies the cytoplasm and inhibits glycolytic enzymes. A decrease in ΔpH by fluoride has a negative effect on the energetic status of the cell because by increasing re-entry of protons across the cell membrane it increases the demand on ATP for acid–base regulation. The final result is increased acidification and starvation stresses on the cell (33).

Self-adhesive resin cements seem to provide promising antibacterial effects. Although an initial low pH value has a great role in antibacterial effects and etching of enamel and dentin, if the low pH lasts for a long time, it might exert negative effects on the adhesion of the cement to dentin (6, 19). Despite the antibacterial properties of fluoride, its activity is still to be elucidated. However, GICs and resin-modified GICs have been reported to be the only materials that release the highest amount of fluoride among the luting agents, but even those products severely reach the inhibitory release level of fluoride (29).

The available data are derived from studies that assessed only a limited number of these cements currently available. Furthermore, bacteria other than *S. mutans* are also responsible for caries, should be investigated in future studies. In addition, long-term clinical antibacterial and anti-cariogenic effects of these materials should be assessed before making general recommendations.

## CONCLUSION

All the evaluated cements exhibited significant antibacterial effects at 1-hour and 24-hour intervals. At 1-hour, G-Cem exhibited higher antibacterial effects than Max-Cem, and Max-Cem showed higher activity than Bis-Cem. At 24-hour interval, Max-Cem showed higher antibacterial effect than G-Cem, and G-Cem showed higher activity than Bis-Cem. At 1-week interval, antibacterial effect of G-Cem and Max-Cem returned to their corresponding control value. However, antibacterial effect of Bis-Cem at this time interval was significantly more than its control value.

## References

[1] Daugela P Oziunas R Zekonis G Antibacterial potential of contemporary dental luting cements. Stomatologija 2008; 10: 16–21. 18493161

[2] Villat C Tran XV Pradelle-Plasse N Ponthiaux P Wenger F Grosgogeat B Impedance methodology: A new way to characterize the setting reaction of dental cements. Dent Mater 2010; 26: 1127–1132. 20728209 10.1016/j.dental.2010.07.013

[3] Korkmaz FM Tuzuner T Baygin O Buruk CK Durkan R Bagis B Antibacterial activity, surface roughness, flexural strength, and solubility of conventional luting cements containing chlorhexidine diacetate/cetrimide mixtures. J Prosthet Dent 2013; 110: 107–115. 23929372 10.1016/S0022-3913(13)60349-2

[4] Feroz S Bhoyar A Khan S Comparative evaluation of antibacterial effect of dental luting cements on *Streptococcus mutans* and *Lactobacillus acidophilus*: An *in vitro* study. J Contemp Dent Pract 2016; 17: 973–977. 27965482

[5] Zhang H Shen Y Ruse ND Haapasalo M Antibacterial activity of endodontic sealers by modified direct contact test against *Enterococcus faecalis*. J Endod 2009; 35: 1051–1055. 19567333 10.1016/j.joen.2009.04.022

[6] Han L Okamoto A Fukushima M Okiji T Evaluation of physical properties and surface degradation of self-adhesive resin cements. Dent Mater J 2007; 26: 906–914. 18203498 10.4012/dmj.26.906

[7] Koo H Sheng J Nguyen PT Marquis RE Co-operative inhibition by fluoride and zinc of glucosyl transferase production and polysaccharide synthesis by mutans streptococci in suspension cultures and biofilms. FEMS Microbiol Lett 2006; 254: 134–140. 16451191 10.1111/j.1574-6968.2005.00018.x

[8] Runnacles P Correr GM Baratto Filho F Gonzaga CC Furuse AY Degree of conversion of a resin cement light-cured through ceramic veneers of different thicknesses and types. Braz Dent J 2014; 25: 38–42. 24789290 10.1590/0103-6440201302200

[9] Aguiar TR Pinto CF Cavalli V Nobre-dos-Santos M Ambrosano GM Mathias P Influence of the curing mode on fluoride ion release of self-adhesive resin luting cements in water or during pH-cycling regimen. Oper Dent 2012; 37: 63–70. 21942239 10.2341/10-328-L

[10] Ebrahimi Chaharom ME Ajami AA Abed Kahnamouei M Jafari Navimipour E Tehranchi P Zand V Antibacterial effect of all-in-one self-etch adhesives on *Enterococcus faecalis*. J Dent Res Dent Clin Dent Prospects 2014; 8: 225–229. 25587384 10.5681/joddd.2014.040 PMC4288912

[11] Huang Q Huang S Liang X Qin W Liu F Lin Z The antibacterial, cytotoxic, and flexural properties of a composite resin containing a quaternary ammonium monomer. J Prosthet Dent 2018; 120: 609–616. 29724549 10.1016/j.prosdent.2017.12.017

[12] Lewinstein I Matalon S Slutzkey S Weiss EI Antibacterial properties of aged dental cements evaluated by direct-contact and agar diffusion tests. J Prosthet Dent 2005; 93: 364–371. 15798687 10.1016/j.prosdent.2005.01.008

[13] Weiss EI Shalhav M Fuss Z Assessment of antibacterial activity of endodontic sealers by a direct contact test. Endod Dent Traumatol 1996; 12: 179–184. 9028181 10.1111/j.1600-9657.1996.tb00511.x

[14] Magalhaes AP Moreira FC Alves DR Estrela CR Estrela C Carriao MS Silver nanoparticles in resin luting cements: Antibacterial and physiochemical properties. J Clin Exp Dent 2016; 8(4): e415–e422. 27703610 10.4317/jced.52983 PMC5045689

[15] Tay FR Pashley DH Aggressiveness of contemporary self-etching systems. I: Depth of penetration beyond dentin smear layers. Dent Mater 2001; 17: 296–308. 11356206 10.1016/s0109-5641(00)00087-7

[16] Piwowarczyk A Bender R Ottl P Lauer HC Longterm bond between dual-polymerizing cementing agents and human hard dental tissue. Dent Mater 2007; 23: 211–217. 16494937 10.1016/j.dental.2006.01.012

[17] Abo-Hamar SE Hiller KA Jung H Federlin M Friedl KH Schmalz G Bond strength of a new universal self-adhesive resin luting cement to dentin and enamel. Clin Oral Investig 2005; 9: 161–167. 10.1007/s00784-005-0308-5 15856343

[18] Zicari F Couthino E De Munck J Poitevin A Scotti R Naert I Bonding effectiveness and sealing ability of fiber-post bonding. Dent Mater 2008; 24: 967–977. 18177701 10.1016/j.dental.2007.11.011

[19] Wang Y Spencer P Continuing etching of an all-in-one adhesive in wet dentin tubules. J Dent Res 2005; 84: 350–354. 15790742 10.1177/154405910508400411

[20] Carvalho AS Cury JA Fluoride release from some dental materials in different solutions. Oper Dent 1999; 24: 14–19. 10337293

[21] Attar N Turgut MD Fluoride release and uptake capacities of fluoride-releasing restorative materials. Oper Dent 2003; 28: 395–402. 12877425

[22] Mukai Y ten Cate JM Remineralization of advanced root dentin lesions *in vitro*. Caries Res 2002; 36: 275–280. 12218277 10.1159/000063924

[23] Levy SM An update on fluorides and fluorosis. J Can Dent Assoc 2003; 69: 286–291. 12734021

[24] Tenuta LM Zamataro CB Del Bel Cury AA Tabchoury CP Cury JA Mechanism of fluoride dentifrice effect on enamel demineralization. Caries Res 2009; 43: 278–285. 19439949 10.1159/000217860

[25] Van Dijken J Kalfas S Litra V Oliveby A Fluoride and mutans streptococci levels in plaque on aged restorations of resin-modified glass lonomer cement, compomer and resin composite. Caries Res 1997; 31: 379–383. 9286522 10.1159/000262422

[26] Hara AT Queiroz CS Freitas PM Giannini M Serra MC Cury JA Fluoride release and secondary caries inhibition by adhesive systems on root dentine. Eur J Oral Sci 2005; 113: 245–250. 15953250 10.1111/j.1600-0722.2005.00214.x

[27] Burke FM Ray NJ McConnell RJ Fluoride-containing restorative materials. Int Dent J 2006; 56: 33–43. 16515011 10.1111/j.1875-595x.2006.tb00072.x

[28] Yoda A Nikaido T Ikeda M Sonoda H Foxton RM Tagami J Effect of curing method and storage condition on fluoride ion release from a fluoride-releasing resin cement. Dent Mater J 2006; 25: 261–266. 16916227 10.4012/dmj.25.261

[29] Wiegand A Buchalla W Attin T Review on fluoride-releasing restorative materials--fluoride release and uptake characteristics, antibacterial activity and influence on caries formation. Dent Mater 2007; 23: 343–362. 16616773 10.1016/j.dental.2006.01.022

[30] Itota T Okamoto M Sato K Nakabo S Nagamine M Torii Y Release and recharge of fluoride by restorative materials. Dent Mater J 1999; 18: 347–353. 10786156 10.4012/dmj.18.347

[31] de Araujo FB Garcia-Godoy F Cury JA Conceicao EN Fluoride release from fluoride-containing materials. Oper Dent 1996; 21: 185–190. 9484170

[32] Li L The biochemistry and physiology of metallic fluoride: action, mechanism, and implications. Crit Rev Oral Biol Med 2003; 14: 100–114. 12764073 10.1177/154411130301400204

[33] Svensater G Sjogreen B Hamilton IR Multiple stress responses in *Streptococcus mutans* and the induction of general and stress-specific proteins. Microbiology 2000; 146: 107–117. 10658657 10.1099/00221287-146-1-107

